# Maternal cardiovascular hemodynamics in normotensive versus preeclamptic pregnancies: a prospective longitudinal study using a noninvasive cardiac system (NICaS™)

**DOI:** 10.1186/s12884-018-1861-7

**Published:** 2018-06-14

**Authors:** Anat Lavie, Maya Ram, Shaul Lev, Yair Blecher, Uri Amikam, Yael Shulman, Tomer Avnon, Eran Weiner, Ariel Many

**Affiliations:** 1 Department of Obstetrics and Gynecology, Lis Hospital, Tel Aviv Sourasky Medical Center, 6 Weizman Street, 6423906 Tel Aviv, Israel; 2 General ICU, Hasharon Hospital, Rabin Medical Center, Petach Tikva, Israel; 3 Department of Obstetrics and Gynecology, Edith Wolfson Medical Center, Holon, Israel; 4 Sackler Faculty of Medicine, Tel Aviv University, Tel Aviv, Israel

**Keywords:** Cardiac output (CO), Cesarean section (CS), Hemodynamics, Preeclampsia with severe features, Pregnancy

## Abstract

**Background:**

Preeclampsia is among the most common medical complications of pregnancy. The clinical utility of invasive hemodynamic monitoring in preeclampsia (e.g., Swan-Ganz catheter) is controversial. Thoracic impedance cardiography (TIC) and Doppler echocardiography are noninvasive techniques but they both have important limitations. NICaS™ (NI Medical, PetachTikva, Israel) is a noninvasive cardiac system for determining cardiac output (CO) that utilizes regional impedance cardiography (RIC) by noninvasively measuring the impedance signal in the periphery. It outperformed any other impedance cardiographic technology and was twice as accurate as TIC.

**Methods:**

We used the NICaS™ system to compare the hemodynamic parameters of women with severe preeclampsia (PET group, *n* = 17) to a cohort of healthy normotensive pregnant women with a singleton pregnancy at term (control group, *n* = 62) (1/2015–6/2015). Heart rate (HR), stroke volume (SV), CO, total peripheral resistance (TPR) and mean arterial pressure (MAP) were measured 15–30 min before CS initiation, immediately after administering spinal anesthesia, immediately after delivery of the fetus and placenta, at the abdominal fascia closure and within 24–36 and 48–72 h postpartum.

**Results:**

The COs before and during the CS were significantly higher in the control group compared to the PET group (*P* < .05), but reached equivalent values within 24–36 h postpartum. CO peaked at delivery of the newborn and the placenta and started to decline afterwards in both groups. The MAP and TPR values were significantly higher in the PET group at all points of assessment except at 48–72 h postpartum when it was still significantly higher for MAP while the TPR only exhibited a higher trend but not statistically significant. The NICaS™ device noninvasively demonstrated low CO and high TPR profiles in the PET group compared to controls.

**Conclusions:**

The immediate postpartum period is accompanied by the most dramatic hemodynamic changes and fluid shifts, during which the parturient should be closely monitored. The NICaS™ device may help the clinician to customize the most optimal management for individual parturients. Our findings require validation by further studies on larger samples.

## Background

Preeclampsia is among the most common medical complications of pregnancy, with an incidence of 4.6% of pregnancies worldwide [1] and is defined as the onset of hypertension with either proteinuria or end-organ dysfunction or both at the second half of pregnancy in a previously normotensive woman. The severe form of the disease includes severe hypertension and signs or symptoms of end-organ injury [2]. Though most affected pregnancies deliver at term, with good outcomes, they carry increased risk for maternal and fetal complications [3, 4].

A major disagreement exists in the literature regarding the hemodynamics of preeclampsia, attributed mainly to variances in the definition and severity of the disease and the techniques used to quantity cardiac output (CO) and blood pressure (BP) [5].

Until the early 1970s, the techniques for measuring cardiac hemodynamics were mostly based upon invasive methods, such as the Swan-Ganz catheter [6–8]. However, the clinical value of invasive hemodynamic monitoring in preeclampsia is debatable [9]. Moreover, right heart catheterization with the Swan-Ganz catheter carries significant risks, including arrhythmias, thrombosis and death [10–12]. The emergence of noninvasive techniques has made it feasible to perform serial measurements of maternal hemodynamics throughout pregnancy. The most widely used techniques include thoracic impedance cardiography (TIC) and Doppler echocardiography, however, they both have important limitations. Doppler echocardiography requires expensive equipment and intensive training of operators. TIC is simpler to use and inexpensive, however the signals used to estimate the stroke volume (SV) are taken from the thoracic area and are exposed to competing signals from the lungs, vena cava and movement of the heart. As a result, the accuracy and reproducibility are limited [13, 14].

In 2010, a noninvasive cardiac system (NICaS™, NI Medical, Petach Tikva, Israel) for determining CO first became available for clinical use. The method utilizes regional impedance cardiography (RIC) by measuring noninvasively the impedance signal in the periphery. The new approach was validated by several studies [14–16], which found that NICaS™ outperformed any other impedance cardiographic technology and was twice as accurate as TIC.

Here, we compare maternal cardiac functional parameters in normotensive versus preeclamptic women undergoing cesarean section (CS) under spinal anesthesia. Establishing the typical range of the parameters associated with preeclampsia would allow the clinician to assess the level of deviation from expected cardiac performance and help to shed light upon the optimal clinical management of individual pathological situations.

## Methods

Ethical approval for the study was granted by the local Institutional Review Board Ethics Committee (decision 0004–15-TLV from 1/2015), and all subjects gave written informed consent. The study population comprised 17 women with a singleton pregnancy complicated by preeclampsia with severe features as defined according to the ACOG criteria [2] who required urgent delivery by CS. Sixty two low-risk women with a singleton pregnancy at term (gestational age ≥ 37 weeks) who were scheduled for elective CS due to obstetric indications (e.g., abnormal fetal presentation, previous CS, narrow pelvis and patient’s request), served as controls. Excluded from the study were women with fetal anomaly, multiple fetuses, chronic hypertensive disorders and cardiovascular disease.

### Study design

This prospective longitudinal observational study was performed at a tertiary medical center. All the women were assessed for hemodynamic changes using the NICaS™ device while lying in the supine position with leftward tilt, as recently described by us [17, 18], and at the following time points: 15–30 min before undergoing a CS (measurement #1), immediately after receiving spinal anesthesia (measurement #2), immediately after delivery of the newborn and the placenta (measurement #3), at the closure of abdominal fascia (measurement #4), within 24–36 h (measurement #5), and 48–72 h postpartum (measurement #6).

### Institutional CS protocol

As previously described by our group [17, 18], according to our institutional protocol every patient planned for elective CS is admitted to the hospital being “nothing per os” (NPO) from midnight prior to the CS. Two hours prior to surgery intravenous hydration is initiated with 1 l of Ringer’s lactate at a rate of 150 ml/hour, which is maintained until 1-h post- CS.

Spinal anesthesia is administered in the sitting position, using a 25-gauge spinal needle inserted at the L3–4 interspace and consisted of fentanyl 25 mcg and hyperbaric bupivacaine 10 mg. Following the delivery of the fetus and placenta, all women receive 10 units of oxytocin given in the remaining 500 ml of Ringer’s lactate. In cases of hemodynamic instability Vasopressors (e.g., noradrenaline and phenylephrine) are used. After the CS, the senior surgeon documents the estimated blood loss.

### Institutional preeclampsia protocol

Medical management of all the severe preeclamptic women was carried out in accordance with the accepted standards in the delivery room, and implemented on the basis of:

- Intravenous (IV) magnesium sulfate for seizure prophylaxis consisting of a 4 g loading dose in 100 ml of saline infusion for 20 min is followed by a maintenance infusion of 2 g/h by controlled infusion pump. Magnesium sulfate is administered until 24 h after delivery.
- Antihypertensive medications are indicated if the BP is 160/110 or higher. First line of treatment is IV bolus doses of labetalol (10–20 mg administered over 2 min) every 20–30 min, up to a total of 220 mg.

### NICaS™ device

**Fig. 1:**
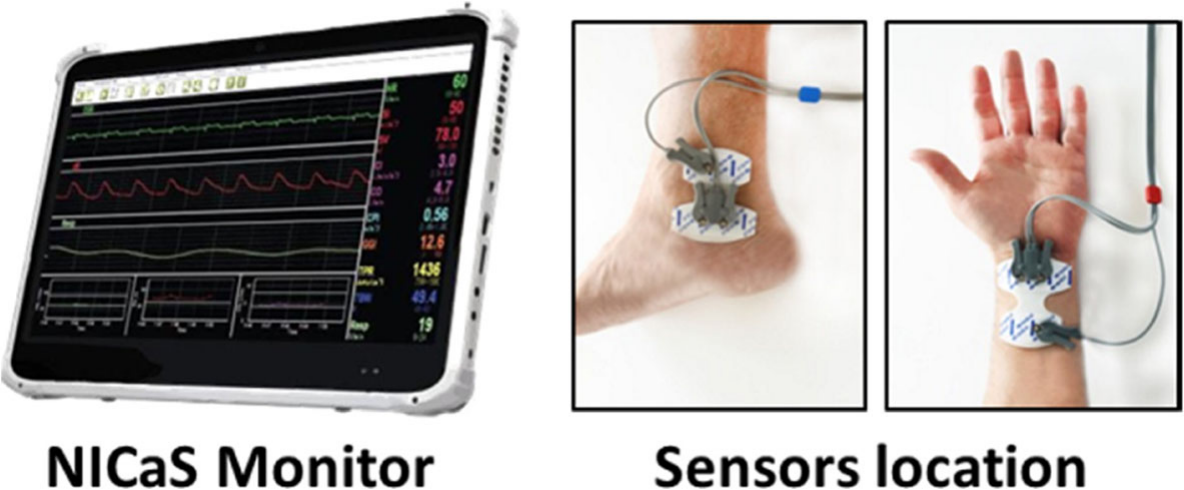
The noninvasive cardiac system (NICaS™) monitor and sensors (Source: NICaSTM, NI Medical Ltd., Petach Tikva, Israel) (with permission of NI Medical)

Two sensors are placed on both wrists or on one wrist and on the contralateral ankle (Fig. 1). SV is calculated by measuring changes of electrical resistance as a result of volume changes of the arterial system {for detailed explanation of the formula please find [14, 17, 18]}.

**Fig. 2:**
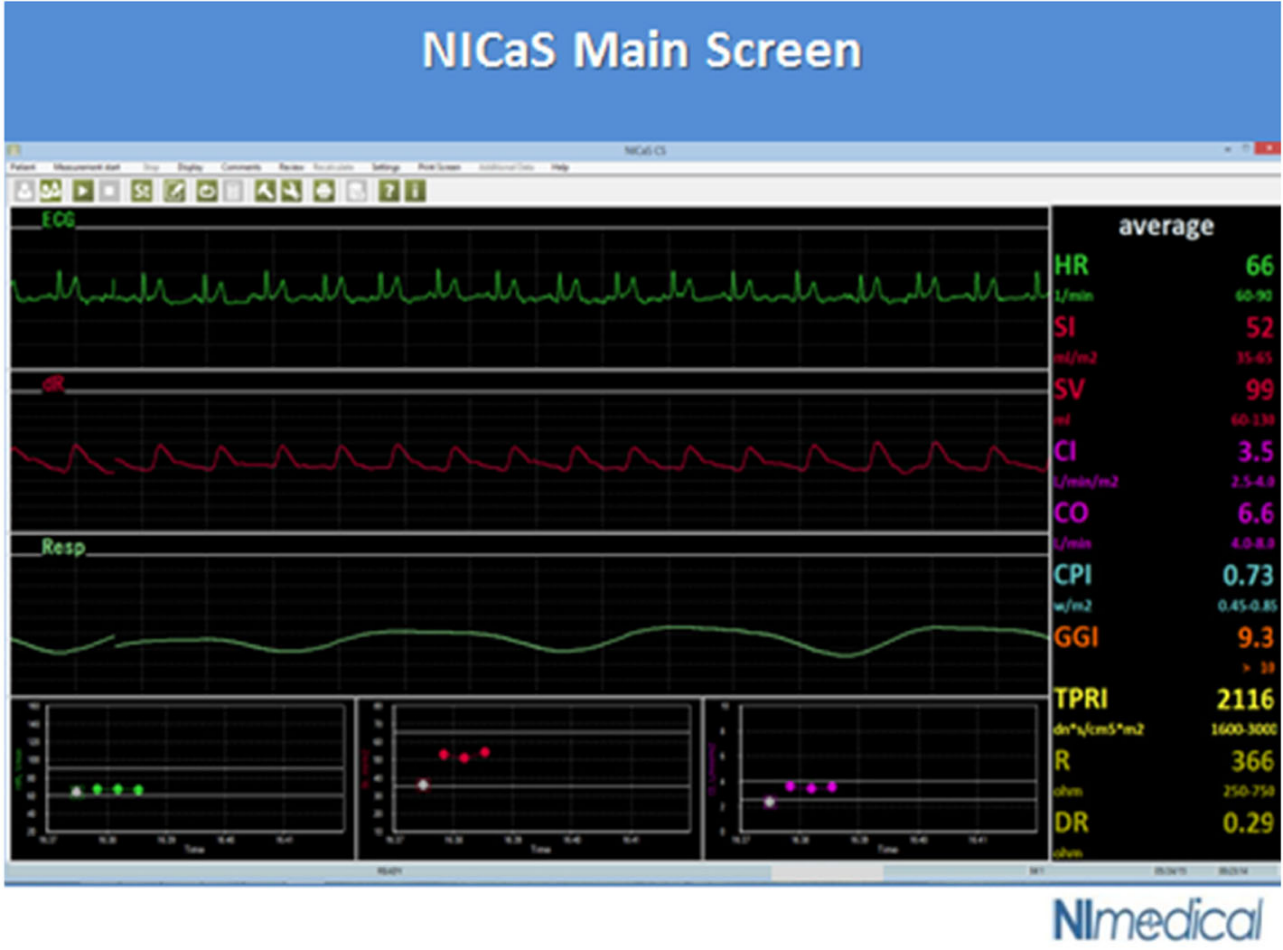
The main screen of the Noninvasive Cardiac System (NICaS™). Top waveform (green) –ECG, middle waveform (red) – whole-body impedance, lower waveform (white) – respiration. Bottom 3 graphs provide trends of HR, SV and CO. Data is displayed on the right. (Source: NICaSTM, NI Medical Ltd., Petach Tikva, Israel) (with permission of NI Medical)

**Fig. 3:**
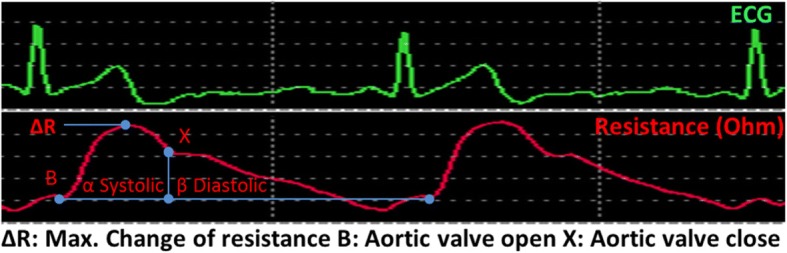
NICaS ECG and Impedance waveforms. SV is calculated base of impedance waveform, HRs calculated based on ECG waveform (Source: NICaSTM, NI Medical Ltd., Petach Tikva, Israel) (with permission of NI Medical)

Heart rate (HR) is measured from a one-channel electrocardiograph, and CO is calculated as CO=SV x HR. With the additional information of systolic blood pressure (SBP) and diastolic blood pressure (DBP) readings by standard cuff, the NICaS™ calculates mean arterial pressure (MAP) as MAP = 2/3DBP + 1/3SBP, and total peripheral resistance (TPR) as TPR = 80xMAP/CO dynexSec/cm^5^. The difference between TPR and systemic vascular resistance (SVR) [SVR = (MAP-CVP)/COx80 dynexSec/ cm^5^], where CVP is the central venous pressure, is that CVP, which is normally very small, is omitted in the TPR calculation. The device meets US FDA requirements for claiming statistical bioequivalence to pulmonary artery catheter-determined CO thermodilution techniques [10]. Hemodynamic, demographic, obstetric and neonatal data were recorded for all cases. Figure 2 depicts the main screen of the system with its measured and calculated parameters and Fig. 3 provides ECG and impedance waveforms produced by the device.

### Statistical analysis

The quantitative data are expressed as medians (first quartile- third quartile) and compared using the Mann-Whitney U test. The statistical significance of the trends between measurements and baseline characteristics of the hemodynamic parameters was calculated by using a mixed model with repeated measures analysis with Kenward-Roger degrees of freedom adjustment and the Tukey-Kramer correction for multiple comparisons using Proc Mixed in SAS Version 9.1.3 (SAS Institute, Inc., Cary, NC, USA). All *P* values < 0.05 were considered significant.

## Results

A total of 64 healthy pregnant women were scheduled for elective CS and fulfilled the inclusion criteria for this study. These women served as the control group. Two withdrawal their consent, and the remaining 62 completed measurements #1 and #5. A technical matter made measurement #2 impossible for two women, and three women were discharged early from the maternity ward, hence lost follow-up for measurement #6. Schedule limitations during the CS did not enable us to complete measurements #3 and #4 for all patients, therefore 36 women completed the former and 31 women the latter. Seventeen additional women who developed preeclampsia with severe features were recruited for the PET group. We included women whose severity of their preeclampsia was based upon criteria other than severe hypertension [4] so that antihypertensive treatment would not confound our results. All 17 women completed measurement #1. One woman was lost to follow-up on for measurements #5 and # 6. Additionally, for technical reasons, most but not all women completed measurements # 2, #3 and # 4.

None of the candidates met the exclusion criteria.

Pre- and intraoperative fluids were given to each study woman, and each received spinal anesthesia at the level of L3–4, without any sequelae. Ten units of oxytocin was administered immediately after the delivery of the fetus and placenta. No other medications (e.g., vasopressors) were given during or after the CS. There was no case of an abnormal estimated blood loss (> 800 ml) and no blood products were required during or after CS. Similarly, no antihypertensive treatment was needed throughout the study. All of the women in the PET group received magnesium sulfate before entering the study as per our institutional protocol.

Table 1 lists the characteristics of each participant. They were all healthy and none was a smoker. Ten control women and four women in the PET group conceived following in vitro fertilization treatments. Six control women who had diet-controlled gestational diabetes mellitus had pregnancy-related complications, as did one woman in the PET group who also had gestational diabetes.

**Table 1:** Patients’ characteristics

Variable^a^	Total	Control group (*n* = 62)	PET group (*n* = 17)	*P* value^b^
Age, years	34.0 (31.0–38.0)	34.5 (31.0–38.0)	33.0 (31.0–38.0)	NS
Pre-gest. BMI	22.8 (20.0–27.0)	22.8 (20.0–26.0)	26.0 (19.8–29.0)	NS
Current BMI	28.3 (26.2–32.1)	28.3 (26.6–31.6)	29.6 (23.7–32.7)	NS
Gest. age, weeks	38.5 (38.1–39.0)	38.7 (38.4–39.0)	32.6 (31.5–35.7)	<.001
Birth weight, kilograms	3.045 (2.622–3.480)	3.220 (2.970–3.540)	1.456 (1.155–1.904)	<.001
Pre-op Hb, gr/dL	12.3 (11.7–13.0)	12.3 (11.7–13.0)	12.4 (11.8–13.0)	NS
Post-op Hb, gr/dL	11.0 (10.2–11.8)	11.0 (10.3–11.8)	11.0 (9.4–11.5)	NS

^a^ median (25th–75th percentiles)

^b^By Mann-Whitney U Test

*NS* non significant, *Pre-gest* pre-gestational, *BMI* body mass index, *Preop* pre-operational, *Hb* hemoglobin, *Post-op* post-operational

**Fig. 4:**
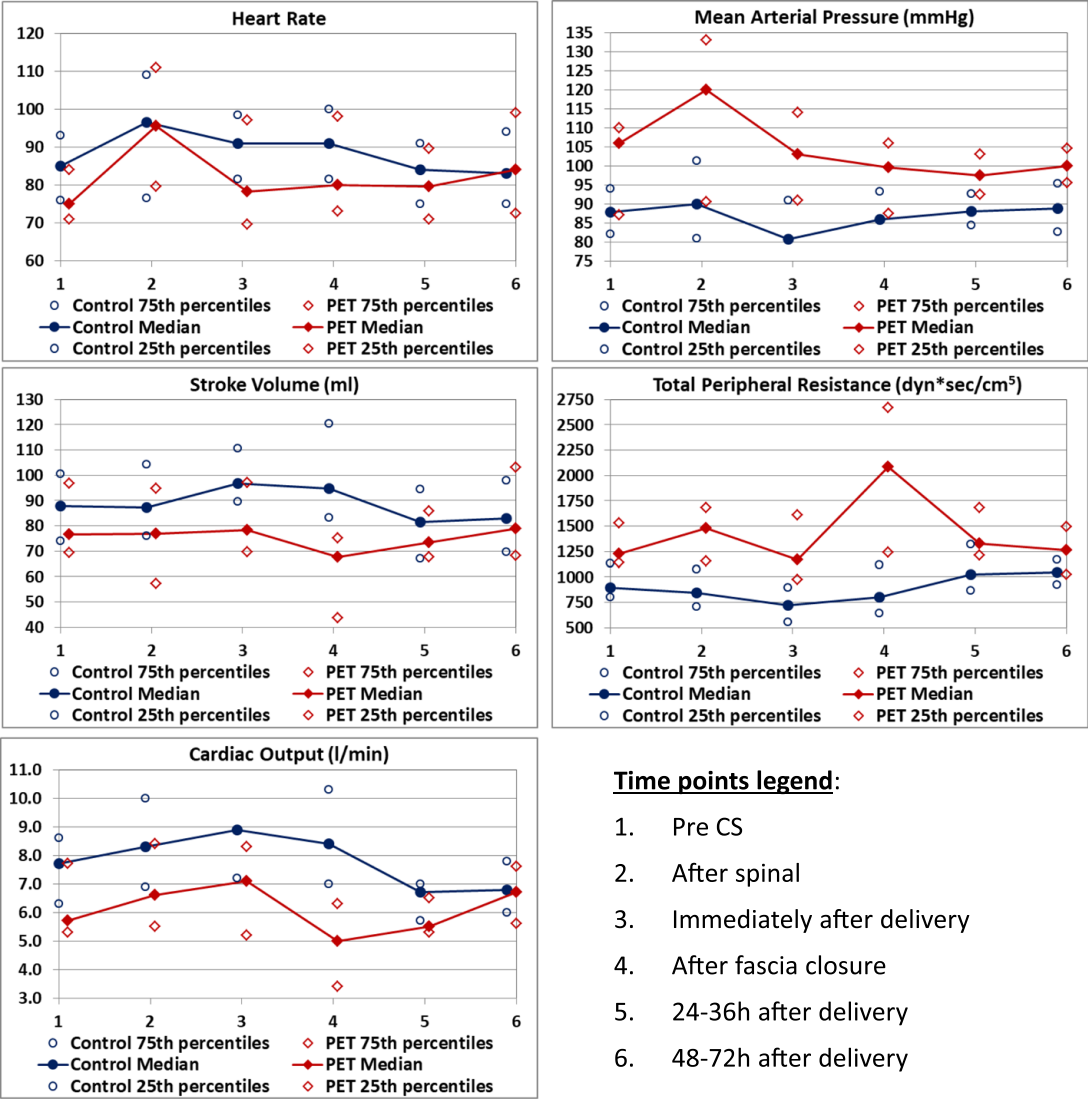
The MAP, TPR, HR, SV, and CO of both groups during the points of assessment: Before CS, after spinal anesthesia, immediately after delivery of the newborn and the placenta, at the time of fascia closure, and within 24–36 h and 48–72 h postpartum

Figure 4 displays the hemodynamic measurements of both groups, and Table 2 compares the hemodynamic parameters between the groups and between successive points of measurements. Comparisons of the hemodynamic parameters of each group between each measurement to baseline (i.e., measurement #1) are listed in Table 3.

**Table 2:** Comparisons of hemodynamic parameters between the groups and between successive points of measurement

	Control group		PET group			
	Median (25th–75th percentiles)	*P* value related to previous	Median (25th–75th percentiles)		*P* value related to previous	*P* value between groups
Before CS	N = 62		N = 17			
MAP (mmHg)	87.8 (82–94)	–	106 (87–110)		–	< 0.001^a^
TPR (dyn × sec/cm5)	896.8 (802.7–1129.7)	–	1227 (1141–1527)		–	0.0042
HR (beats/min)	85 (76–93)	–	75 (71–84)		–	NS
SV (ml)	87.7 (74–100.6)	–	76.6 (69.4–96.8)		–	NS
CO (l/min)	7.7 (6.3–8.6)	–	5.7 (5.3–7.7)		–	0.0331
After spinal anesthesia	*N* = 60		*N* = 12		–	
MAP (mmHg)	90 (81–101.3)	NS	120 (90.5–133)		0.0027	< 0.001^a^
TPR (dyn × sec/cm5)	847.3 (703.5–1074.4)	NS	1477 (1154–1682)		NS	< 0.001^a^
HR (beats/min)	96.5 (76.5–109)	< 0.001^a^	95.5 (79.5–111)		0.0033	NS
SV (ml)	87.1 (76.1–104.3)	NS	76.9 (57.2–94.8)		NS	0.0254
CO (l/min)	8.3 (6.9–10)	0.0075	6.6 (5.5–8.4)		NS	0.0107
Immediately post-delivery	*N* = 36		*N* = 13			
MAP (mmHg)	80.8 (70.5–91)	< 0.001^a^	103 (91–114)		0.0308	< 0.001^a^
TPR (dyn × sec/cm5)	717.9 (558.3–897.3)	0.0339	1166 (972–1606)		NS	< 0.001^a^
HR (beats/min)	91 (81.5–98.5)	NS	84 (77–91)		NS	NS
SV (ml)	96.9 (89.6–110.5)	0.0048	78.3 (69.6–97)		NS	0.0102
CO (l/min)	8.9 (7.2–11.3)	NS	7.1 (5.2–8.3)		NS	0.0054
After fascia closure	*N* = 31		N = 12			
MAP (mmHg)	86 (71.7–93.3)	NS	99.5 (87.5–106)		NS	0.0033
TPR (dyn × sec/cm5)	802.8 (644.8–1115.4)	0.0273	2087 (1240–2671)		< 0.001^a^	< 0.001^a^
HR (beats/min)	91 (81.5–100)	NS	80 (73–98)		NS	NS
SV (ml)	94.7 (83.2–120.3)	NS	67.6 (43.6–75.2)		0.0039	< 0.001^a^
CO (l/min)	8.4 (7–10.3)	NS	5 (3.4–6.3)		0.0083	< 0.001^a^
Within 24–36 h postpartum	N = 62		*N* = 16			
MAP (mmHg)	88 (84.3–92.7)	NS	97.5 (92.5–103)		NS	0.015
TPR (dyn × sec/cm5)	1023.7 (863.5–1316.9)	NS	1326 (1211–1681)		< 0.001^a^	0.0021
HR (beats/min)	84 (75–91)	NS	79.5 (71–89.5)		NS	NS
SV (ml)	81.6 (67–94.5)	< 0.001^a^	73.4 (67.7–85.8)		NS	NS
CO (l/min)	6.7 (5.7–8)	< 0.001^a^	5.5 (5.3–6.5)		NS	NS
Within 48–72 h postpartum	*N* = 59		N = 16			
MAP (mmHg)	88.7 (82.7–95.3)	NS	100.5 (95.5–104.5)		NS	< 0.001^a^
TPR (dyn × sec/cm5)	1043.7 (926.5–1168.4)	NS	1261 (1028–1494)		NS	NS
HR (beats/min)	83 (75–94)	NS	84 (72.5–99)		NS	NS
SV (ml)	82.9 (69.5–98)	NS	79 (68.1–103)		NS	NS
CO (l/min)	6.8 (6–7.8)	NS	6.7 (5.6–7.6)		NS	NS

^a^ Significance was also found after adjusted for multiple comparisons

*P*- values are given when 0.001 < *P* < 0.05

*NS* not significant, *N* number of women, *CS* cesarean section, *MAP* mean arterial pressure, *TPR* total peripheral resistance, *HR* heart rate, *SV* stroke volume, *CO* cardiac output

**Table 3:** Groups' comparisons of the hemodynamic parameters between each measurement to baseline^a^

Parameter	Pre-CS	After spinal anesthesia	*P* value after spinal anesthesia vs. pre-CS	Immediately after delivery	*P-*value immediately after delivery vs. pre-CS	After fascia closure	*P* value after fascia closure vs. pre-CS	+24h after delivery	*P-*value +24h after delivery vs. pre-CS	+48h after delivery	*P-*value +48h after delivery vs. Pre-CS
Control group- comparisons of the hemodynamic parameters between each measurement to baseline^a^											
No. of women	62	60		36		31		62		59	
MAP (mmHg)	87.8 (82-94)	90 (81-101.3)	NS	80.8 (70.5-91)	0.0113	86 (71.7-93.3)	NS	88 (84.3-92.7)	NS	88.7 (82.7-95.3)	NS
HR (beat/min)	85 (76-93)	96.5 (76.5-109)	<0.001^*^	91 (81.5-98.5)	0.0483	91 (81.5-100)	NS	84 (75-91)	NS	83 (75-94)	NS
SV (ml/beat)	87.7 (74-100.6)	87.1 (76.1-104.3)	NS	96.9 (89.6-110.5)	0.0039	94.7 (83.2-120.3)	0.0241	81.6 (67-94.5)	0.0345	82.9 (69.5-98)	NS
CO (l/min)	7.7 (6.3-8.6)	8.3 (6.9-10)	0.0075	8.9 (7.2-11.3)	<0.001^*^	8.4 (7-10.3)	0.0113	6.7 (5.7-8)	0.05	6.8 (6-7.8)	NS
TPR (dyne*sec/cm^5^)	896.8 (802.7-1129.7)	847.3 (703.5-1074.4)	NS	717.9 (558.3-897.3)	0.0026	802.8 (644.8-1115.4)	NS	1023.7 (863.5-1316.9)	<0.001	1043.7 (926.5-1168.4)	NS
PET group- comparisons of the hemodynamic parameters between each measurement to baseline^a^											
No. of women	17	12		13		12		16		16	
MAP (mmHg)	106 (87-110)	120 (90.5-133)	0.0027	103 (91-114)	NS	99.5 (87.5-106)	NS	97.5 (92.5-103)	NS	100.5 (95.5-104.5)	NS
HR (beat/min)	75 (71-84)	95.5 (79.5-111)	0.0033	84 (77-91)	NS	80 (73-98)	NS	79.5 (71-89.5)	NS	84 (72.5-99)	NS
SV (ml/beat)	76.6 (69.4-96.8)	76.9 (57.2-94.8)	NS	78.3 (69.6-97)	NS	67.6 (43.6-75.2)	0.0064	73.4 (67.7-85.8)	NS	79 (68.1-103)	NS

^a^Data are given as median (25th-75th percentiles)

*Significance was also found after adjustment for multiple comparisons

*P*-values are given when 0.001 < *P* <0.05

Abbreviations: NS, non-significant; CS, cesarean section; MAP, mean arterial pressure; HR, heart rate; SV, stroke volume; CO, cardiac output; TPR, total peripheral resistance

MAP was significantly higher in the PET group compared to the control group (*P* < 0.05) at all points of assessment. When adjusted for multiple comparisons, there were significance differences for measurements #1, #2, #3 and #6 (*P* ≤ .001). TPR was significantly higher in the PET group compared to the control group (*P* < .05) at measurements #1–5 of assessment. When adjusted for multiple comparisons, significance was found for measurements #2–4 (*P* < .001).

No significant changes were observed in SV and TPR following spinal anesthesia in both groups. Spinal anesthesia caused an increase in HR and CO in the control group. As well, it caused an increase in MAP and HR in the PET group (*P* < .05).

In the control group, both the TPR and MAP reached their lowest values immediately after delivery of the newborn and placenta (*P* < .05). The MAP fully returned to its preoperative values by the time of fascia closure, while the TPR gradually recovered throughout fascia closure until reaching a value of 14% higher than baseline at 24–36 h after delivery.

The PET group demonstrated a dramatic rise in MAP at the beginning of the CS, which peaked after spinal anesthesia and then dropped. Immediately after delivery, the values stabilized and returned to those recorded prior to the CS. The TPR remained constant from the beginning of the CS until immediately after the delivery, then rose dramatically until after the closure of abdominal fascia when it subsequently dropped, reaching preoperative values by 24 h postpartum.

The CO was significantly lower in the PET group compared to the control group (*P* < 0.05) at measurements #1–4. When adjusted for multiple comparisons, only point #4 was significantly lower in PET group, i.e. 5 L/min (interquartile range: 3.4–6.3) compared to 8.4 L/min (interquartile range: 7–10.3) for the controls (*P* < .001). There were no significant group differences in HR at all points of assessment. Consequently, the differences in CO between the groups was attributed to the differences in SVs, which were significantly lower in the PET group compared to the control group at points #2-#4 of assessment. When adjusted for multiple comparisons, the SV was significantly lower only at point #4, where it was 67.6 ml (interquartile range: 43.6–75.2) in the PET group compared to 94.7 ml (interquartile range: 83.2–120.3) in the control group (*P* < .001).

The CO of the women in the control group rose from the pre-CS values throughout the CS and peaked immediately after delivery. This peak contributed to the 10.5% post-delivery increase in SV (*P* = 0.0039) and overshadowed the 5.5% post-delivery decrease in HR. The CO continued to gradually decline until it reached the lowest point, i.e., 13% lower than baseline values at 24–36 h post-delivery (*P* < .001). A drop in SV was considered as being mainly responsible for this decline.

The CO values in the PET group showed a rising trend throughout the CS, and then dropped after the delivery of the newborn and extrusion of the placenta until reaching nadir at the fascia closure (*P* = 0.0083). This decline was mainly due to decline of the SV rather than the HR. The CO then started to rise gradually until 48–72 h post partum.

Within 24–36 h postpartum, the CO in both groups had reached similar values (5.5–6.7 L/min), which were lower than the control group’s pre-CS values and equivalent to the PET group’s pre-CS values. None of the measured variables (CO, MAP, TPR, HR, and SV) differed significantly between the two groups in the measurements taken between 24 and 36 and 48–72 h postpartum.

The significant between-group interactions that had been observed in the hemodynamic patterns and their points of assessment persisted after adjustment for the demographic parameters of age, gestational week, pre-gestational body mass index (BMI), neonatal weight, and hemoglobin difference between before and after CS (multivariable analyses).

## Discussion

Understanding maternal hemodynamics is of great importance in pregnancy follow-up, delivery management and prevention and treatment of obstetrical complications. BP is the first hemodynamic parameter to be evaluated when an unstable state develops. BP is the product of CO and TPR, and the benefit of the NICaS™ system is the ability to differentiate between these two components.

In this prospective observational study, we aimed to assess hemodynamic parameters in severely preeclamptic women compared to healthy ones, in order to characterize the hemodynamic parameters that represent the most severe form of preeclampsia. Our rational was that if we compared women with only mild disease, the differences in hemodynamics would be less noticeable and less characterizable. Moreover, we chose to explore the nature of hemodynamics of women undergoing a CS rather than vaginal delivery, without masking it with the effect of contractions and pushing.

The main findings of our study were: (1) the hemodynamic profiles of the PET group were characterized by high MAP and TPR values and low CO values compared to the control group; (2) the CO in both groups was primarily impacted by changes in SV rather than in HR; (3) the CO in both groups peaked at the delivery of the newborn and the placenta and started to decline afterwards continuing to decline after fascia closure in the control group while increasing in the PET group; (4) there were no differences in the findings of the assessments of both groups made at the 24–36 and 48–72 h postpartum time points; (5) there was no correlation between the selected demographic parameters and the trends in the hemodynamic patterns of all the subjects.

The hemodynamics of preeclampsia have been traditionally characterized by increased vascular resistance, reduced perfusion and high CO [9, 19, 20]. Data gathered from researchers on preeclamptic women in recent years were inconsistent in characterizing the hemodynamic profile of the condition. Nevertheless, the data suggests that as the severity of the condition increases, there is a trend to a change from a high CO profile to a high resistance one [21], and that anti-hypertensive treatment results in a shift from low to high CO [5, 22]. Accordingly, in our study, the PET group, which demonstrated a low CO profile, presented with clinically evident symptoms that had reached a severe level, and they had not been treated with anti-hypertensives. Other investigators reported similar findings [23–26]. A few studies [27–30] showed that remote from term, the hemodynamics of women destined to become preeclamptic were characterized by increased CO and compensatory vasodilatation. Of note, the women in these studies were recruited prior to the onset of the clinical phase of preeclampsia.

Alterations of the CO in both of our study groups were mainly attributed to changes in SV rather than in HR. The HR measurements were equivalent for both groups for all successive measurements in each group. This finding contradicts the reports of others who observed that alterations in HR influence CO in both normotensive and preeclamptic women [23, 26]. We found that CO values in the third trimester and following delivery are mainly controlled by the SV component, as was established by Gordon et al. [31] and by Robson and associates [32].

Our study results suggest that the most dramatic time point in which alterations in hemodynamic parameters take place is the time of delivery. These findings are consistent with those of others [26, 33–35]. Some authors have suggested that changes in CO around the time of delivery result from the relief of caval compression together with an increase in venous return (due to auto-transfusion of blood from the choriodecidual space into the central circulation as the uterus contracts after delivery) [36]. Conversely, other authors have attributed the rise in SV during the first minutes after cesarean delivery to the prominent vasodilatory effects of oxytocin, and stated that the physiological changes in SV are small and nearly negligible. They noticed a lack of increase in SV in women who received placebo as opposed to oxytocin after the delivery of the baby during a CS, which they believe challenges the hypothesis that uterine contraction causes autotransfusion of uterine blood that leads to an increase in preload [37]. Since all participants in our study received oxytocin routinely after the expulsion of fetus and placenta we cannot relate to this interesting debate.

The short-lived increase in CO in the control group at the time of delivery could also be a compensatory mechanism for the significant decrease in TPR associated with the removal of the placenta, which plays a major resistance role. The values of both the MAP and TPR of the control women decreased immediately after delivery. The drop in TPR, however, was even higher (26% compared to 8%), yielding the net effect of a higher CO (TPR = 80xMAP/CO ↔ CO = 80xMAP/TPR). The influence of the uterine contraction was subsequently apparent (and not earlier as in the auto-transfusion theory). The closure of the vasculature at the placental bed site by uterine contractions resulted in the elevation of TPR with a concomitant increase in afterload, and led to a decrease in CO.

In the PET group, however, the observed hemodynamic trends were quite different, with the TPR rising dramatically after delivery, possibly secondarily to the release of mediators from the preeclamptic placenta, and declining only after fascia closure. At the same time, CO acted in opposite directions (dropping after delivery and beginning to increase at the fascia closure) compensating for the disturbance in the vascular tone, preventing BP from rising further. Similarly to Sibai et al. [5], our findings suggest that the problem in preeclampsia is one of a systemic vascular resistance which is inappropriately high for the level of CO.

NICaS™ pre-CS measurements of our control group’s TPR and MAP are similar to the findings of Clark el al. [38]. Specifically, our pre-CS MAP values are similar to their non-pregnant ones, while our TPR values are lower than their non-pregnant ones. The cause for a reduced TPR in the third trimester is most probably progesterone-mediated smooth muscle relaxation. The exact mechanism, however, is unclear, and the suggested mediators include NO, angiotensin 2 and RAAS [39, 40]. As noted earlier, the control group’s TPR and MAP dropped to nadir after the fetus and placenta had been delivered. They subsequently increased gradually until they had reached a plateau. By 24–36 h postpartum, MAP reached pre-CS values and TPR reached significantly higher pre-CS values. The hemodynamic parameters at 48–72 h postpartum (#6) were equal to those of 24–36 h (#5). It is highly likely that these findings are indicative of a return to the expected MAP and TPR in the non-pregnant population, and serve to further validate the reliability of the NICaS™ device.

In pregnancies complicated by preeclampsia, however, TPR at third trimester is not lower than pre-pregnancy values. One suggested mechanism is loss of refractory to the vasoconstrictive effect of angiotensin II [40]. The fact that the TPR values in the PET group were significantly lower than those measured closer to delivery (#4) but still significantly higher than the control group by 24–36 h postpartum, and that these differences were already insignificant by 48–72 h postpartum indicates a rapid recovery of the heart and vasculature in a population of healthy young women. Indeed, the steadiness of hemodynamic measurements observed between 48 and 72 h after delivery in both groups is in accordance with some studies [30, 41], and in opposition to others [34].

The effect of spinal anesthesia and oxytocin on maternal hemodynamics are detailed here, as previously described by our group studies on low risk [17] and twins [18] pregnancies. Hypotension is a familiar effect of spinal anesthesia, induced by sympathetic nervous system block [42, 43] and results in a decrease in SV, CO and TPR. Pretreatment with fluid administration before spinal anesthesia became a universal prophylaxis therapy for this phenomenon [44, 45]. In our study, spinal anesthesia increased HR in both groups but did not affect TPR or SV in either group, probably due to the universal pre-treatment with 500 ml of Ringer lactate. This is consistent with the finding of Toptas and colleagues [42].

Oxytocin’s ability to decrease TPR and increase HR, SV and CO has been demonstrated in several studies [37, 46, 47]. However, both our study and control groups showed the opposite effect following its administration at the time of delivery (measurement #3). As such, the hemodynamic patterns observed throughout measurement #4 could only be explained by the contraction of the uterus and its influence on peripheral vascular resistance. An alternative suggestion by Weis et al. [46] was that oxytocin produced no circulatory changes when given as a dilute solution. The fact that, unlike Desai et al. [48] we found no significant correlation between CO and demographic variables, e.g., fetal birth weight and maternal height and weight, further supports our results, which were derived solely from the stage of labor and the presence or absence of preeclampsia.

Importantly, our study addresses several issues. First, the introduction of the noninvasive NICaS™ technique allowed us a simple and continuous measurements of the cardiovascular parameters. Second, we were able to recruit a relatively large control group. Third, participants were free of any background morbidity or treatment for hypertension, which could have biased the results. Lastly, some of our data differ from previously published studies thus open new avenues in hemodynamic fields.

Our study has some limitations that should be mentioned. It describes hemodynamic changes during CS under spinal anesthesia and does not reflect the effect of hemodynamic changes taking place during vaginal delivery. Other studies, however, have shown that these are quite comparable [33, 49, 50]. Second, the average BMI in our study reflects the healthy average population in our medical center, nonetheless higher BMI might affect the hemodynamic results. Additionally, we controlled for possible confounders (strict protocols for anesthesia, oxytocin and fluids administration, and documentation of blood loss). Still, other factors that might have influenced the result (magnesium sulfate treatment, for example) could not be controlled for. Yet, all women in the PET group received magnesium sulfate by a strict protocol and at the same time (before entering the study), which reduce its confounding effect to a minimum. Lastly, the sample size of the PET group is small and at both groups there are several incomplete measurements. We chose the mixed model analysis to overcome the disadvantages of the small sample size of the PET group and the incompleteness of the measurements. Mixed models are the preferable method of analysis of repeatedly measured outcomes when there are missing data, the repeated measures are irregularly spaced over time, and the sample is small [51, 52]. However, we do recommend repeating the analysis in the future with a larger sample to see if the trend will remain.

## Conclusions

Knowledge of normal hemodynamic values during various stages of pregnancy and the postpartum period is feasible. It might assist clinicians in assessing patient’s deviation from expected cardiac performance and aid monitoring and treating medical complications of pregnancy, such as preeclampsia.

We demonstrated that untreated women with severe preeclampsia have a low CO and a high TPR profile. Because of the dramatic reversal in hemodynamic parameters at the time of delivery itself, if there is one moment which should be selected for aggressive monitoring, it would be the time of delivery. Our study might assist clinicians in assessing the level of patient deviation from expected cardiac performance. The NICaS™ can differentiate between the CO and the TPR components during blood pressure monitoring and thereby aid and enable a suitable treatment, antihypertensive versus volume overload for example. Further research is warranted in order to evaluate the value of NICaS™ in assessing other abnormal medical conditions of pregnancy.

## Abbreviations

BP: Blood pressure
CO: Cardiac output
CS: Cesarean section
HR: Heart rate
MAP: Mean arterial pressure
PET: Preeclampsia toxemia
RIC: Regional impedance cardiography
SV: Stroke volume
TIC: Thoracic impedance cardiography
TPR: Total peripheral resistance
